# The Spread and Development of Psychodrama in Mainland China

**DOI:** 10.3389/fpsyg.2018.01368

**Published:** 2018-08-20

**Authors:** Zhi-qin Sang, Hao-ming Huang, Anastasiia Benko, Yin Wu

**Affiliations:** Department of Psychology, School of Social and Behavioral Sciences, Nanjing University, Nanjing, China

**Keywords:** psychodrama, mainland China, localization, history, campus psychodrama

## Abstract

The history of the popularization and research of psychodrama in the west has spanned more than 80 years since it was founded by J. L. Moreno in the 1930s. However, it was only in the 1990s that psychodrama was systematically introduced in mainland China. The historical process of the spread and development of psychodrama in China is complex; therefore, this study approached it from the perspectives of space and time and theoretical development. Considering four events as the critical time points, the history of psychodrama in China can be divided into four periods from a spatiotemporal perspective: pre-contact period, preparatory period, period of prosperity, and the period of new development. Based on the theoretical classification, three major branches of psychodrama in mainland China are represented by Gong Shu’s Yi Shu psychodrama, Katherine Hudgins’ therapeutic spiral model, and campus psychodrama developed by Chinese psychologists.

## Introduction

Psychodrama (PD) is an action-based group method to explore psychological and social problems. The use of PD techniques allows participants to explore their past, present, or future problems or situations, not by the participants’ simple narrative, but by playing related events in daily life. The dramatic enactment not only shows the explicit external behavior but also more importantly explores the inner world of clients, such as some unsaid thoughts and feelings, unmanifested conflicts, the possible thoughts and feelings of others in imagination and the foresight of future possibilities, etc. It provides opportunities for self-reflection (Blatner, 2000). Jacob Levy Moreno founded PD in the 1930s. And the first time that the Chinese psychologist contacted PD could be traced to 1940s, but the systematical introduction of PD to mainland China started from 1990s. The introduction and popularization of PD techniques and training practices have undergone a long and complicated process. Therefore, this study approached it from the perspectives of space and time and theoretical development.

## History and Development of Psychodrama and Related Research

Psychodrama was founded by psychiatrist J. L. Moreno in the 1930s. In 1921, he began to explore “impromptu shows”, which marked the birth of PD. The basic techniques of PD were established from 1936 to 1940 (Blatner, 2000). Philosophically, J. L. Moreno rejects complete psychological determinism, as it is supported by Freud, in favor of his concept of spontaneity, which he considers as outside of determinism. Encouragement of free and spontaneous expression in inhibited patients can be therapeutic, as can the spontaneous dramatic solution of conflicting drives (Jenkins, 1947). He defined PD as “the science which explores the ‘truth’ by dramatic methods. It deals with inter-personal relations and private worlds”. (Moreno, 1953, p. 81). After J. L. Moreno’s death in 1974, his wife Zerka Moreno published numerous PD-related works according to her husband’s will and helped popularize PD techniques worldwide, becoming the global academic leader of PD (Li, 1995) until her death in 2016. And by leading numerous workshops, she contributed to the popularization of PD in Taiwan and mainland China.

Taking into account the establishment of internationally recognized and influential PD-related academic institutions, the following are the turning points in the history of PD. In 1942, J. L. Moreno established the American Society for Group Psychotherapy and Psychodrama, which was the first professional association for group psychotherapists (Blatner, 2000). In 1964, the first international conference on psychodrama was held in Paris. And then, the psychodramatists regularly held international meetings in Latin America, Europe, and Japan (Hare and Hare, 2004). In 1975, the American Board of Examiners in Psychodrama, Sociometry, and Group Psychotherapy (ABEPSGP) was established, and two levels of certification were determined: certified practitioner (CP) and trainer, educator, and practitioner (TEP). Since then, independent PD qualifications have gradually been established (Buchanan, 2009).

From the perspective of international academic research in psychology, PD theory and methodology has been researched since the 1950s. The literature predominantly consists of quantitative analyses of the effectiveness of PD treatment for specific populations and reviews of theoretical innovations. The quantitative studies mostly involve controlled clinical trials that examine the effectiveness of psychotherapy on patients with mental disorders, such as eating disorders (Pellicciari et al., 2013), drug addiction (Yablonsky, 1959; Somov, 2008), and depression (Ackerman and Ackerman, 1962; Nazar et al., 2014). The reviews of theoretical innovations have primarily focused on the mechanisms underlying the effectiveness of PD and the clarification of its theoretical structure (Kipper, 1978; D’Amato and Dean, 1988; Manzella and Yablonsky, 1991; Blatner, 1997, 2000; Kipper and Hundal, 2003)

## History of Psychodrama in Mainland China

The history of China’s access to PD can be traced back to the early 1940s when a Chinese medical psychologist, Ding Zan, visited J. L. Moreno’s studio in the United States and attempted to promote Moreno’s PD therapy theory in China (Ding, 1948). However, due to various reasons, this early connection was interrupted for over 30 years. In the Great Proletarian Cultural Revolution, psychology was stigmatized as bourgeois pseudoscience, and the development of psychology as a discipline was suspended (The Executive Committee of the Chinese Psychological Society, 1982). In 1974, a Taiwanese psychiatrist Chen Zhuzhang and others founded the first Sub-department of Psychodrama in the Department of Psychiatry of Taiwan National University, marking the official beginning of PD development in the Chinese-speaking region (Lai, 2013). In the 1980s, PD was introduced in mainland China for the second time (Chen, 1982). In the late 1990s, a group of psychodramatists from the United States and Taiwan came to mainland China to further promote practical training in PD.

Through interviews with two psychologists who conducted training and research on PD in China, we distinguished four turning points in the history and development of PD: (1) Ding Zan discovered PD in J. L. Moreno’s studio in the United States in 1948. (2) In the 1980s, PD was introduced to the Chinese psychiatric community by Chen and Li, as a type of psychotherapy for the treatment of mental disorders. (3) From 2004 to 2009, Gong Shu, Katherine Hudgins, Pam and Rory Remer, Lai Nianhua, and other PD Trainers organized various types of PD training in Nanjing and other locations. (4) In 2014, the Division of Group Counseling and Group Therapy of the Chinese Association for Mental Health (CAMH) established the Group of Psychodrama. This group of PD is an organization aimed to promote the development of professional training and research of psychodrama in China. These four turning points divide the history of PD in China into four periods: pre-contact period, preparatory period, period of prosperity, and the period of new development (Sang, 2015).

### Pre-contact Period

The first discussions regarding PD in China can be traced back to the 1940s. At that time, Ding Zan was studying for his master’s degree in psychology at the University of Chicago. In 1948, he spent a month in New York, where he participated in a PD group led by J. L. Moreno and also attended several lectures on PD. Accompanied by Moreno, he also visited a New York drama psychotherapy clinic, PD theater, and Beacon Hill Sanitarium. Later, Ding attempted to introduce PD theory to mainland China. In December 1948, four articles titled “Psychodrama Therapy: Memories of the Training from New York” written by Ding were serialized in Ta Kung Pao which is the oldest active Chinese language newspaper in China (Ding, 1948). Because of the political changes in mainland China, the development of psychology as a discipline was suspended shortly after the founding of the People’s Republic of China, and academic ties initiated by Ding and Moreno were severed for more than three decades.

### Preparatory Period

The preparatory period lasted from the mid-1980s to 2004. In the 1980s, PD as a type of psychotherapy for the treatment of mental disorders was introduced to the Chinese psychiatric community (Chen, 1982; Li, 1988). The earliest paper on the theory of PD was a literature review titled “Psychodrama Therapy”, which was published in 1988 by Li in the journal Shanghai Psychiatry. And the first article in the CNKI (China Knowledge Resource Integrated Database) to discuss PD in mainland China was published in 1982, which was a review article, titled “Introduction to Several Ways of Healing Mental Illnesses” by Chen and published in the Chinese journal Psychological Science.

However, at that time, Chinese psychology and psychiatry specialists did not pay much attention to this type of therapy, and it was not practiced. This continued until July 1995, when Gong Shu, a Chinese-American psychologist, visited Nanjing Brain Hospital to conduct a symposium on expressive psychotherapy and traditional Chinese therapeutic methods. Psychotherapy practitioners and researchers working in the field in China discovered that the theories of psychotherapy, which originated in the West, integrated well with their culture and manner of thinking. Gong Shu’s theory and the techniques of Yi Shu PD were introduced to mainland Chinese scholars. At this point, the concept of PD began to attract the attention of psychotherapists in China and was included in the Chinese Encyclopedia of Psychology (1995) in the same year. In October 1997, the co-founder of PD, Zerka Moreno, visited Nanjing and Beijing, and conducted seminars on PD with Gong Shu. At this point, not only had the psychologists and scholars in China begun to address the concept of PD therapy, but also international academic PD institutions, which began to establish contact with the Chinese academia. Therefore, this period can be considered the preparatory period of PD development in mainland China. At this stage, the psychology community in mainland China began to accept the concept of PD, and understand and study PD theory and techniques on a small scale. However, little efforts were taken at that time to conduct independent research and popularize PD.

### Period of Prosperity

After 1997, although the theory and practice of PD gradually became more popularized in mainland China, it was mostly chaotic and disorganized, lacking an authoritative organization or group to provide professional training to mental health professionals. Moreover, there was a lack of international psychodramatists visiting mainland China to share their knowledge. This situation gradually began to change after 2004. PD training sessions began to be conducted by authoritative specialists. Gong Shu held the first and second International Psychodrama Training of Zerka Moreno events at Nanjing University in 2004. Subsequently, annual PD training sessions or workshops were organized in Nanjing, Suzhou, and other cities. The successful organization of the first International Symposium on Expressive Psychotherapy and Psychodrama in Suzhou in 2007 marked the beginning of the official establishment of PD training and research in China. On March 6, 2010, the International Research Center for Yi Shu Body and Mind Therapy was established at the Faculty of Education of Suzhou University, which also marked the birth of the first formal PD-related research and promotion institution in China. Jiangsu Province became the academic center of PD therapy promotion in China.

Furthermore, 2004 is a crucial point in time also from the perspective of communication with international PD scholars. It was during this year that Gong Shu returned to Nanjing to conduct PD training. After that, Katherine Hudgins, the founder of the therapeutic spiral model (TSM) of PD in the United States, also visited Nanjing in 2004 and conducted numerous training sessions on TSM at Nanjing University. Following her visit to Nanjing, she later conducted training sessions or lectures in Shanghai, Jinan, Beijing, Chongqing, Zhuhai, and other locations. And the training subject included basic PD skills, TSM therapy for attempted suicide survivors and eating disorder clients, and its application in prison management and EAP. Hudgins also set up a TSM working group in mainland China to train a professional TSM team. Starting in 2009, Pam and Rory Remer, psychodramatists and professors at the University of Kentucky, United States, also visited China for PD-related training.

In addition, Professor Lai Nianhua and other new generation psychodramatists from Taiwan began to conduct long-term continual PD training sessions in mainland China. At the same time, all types of PD theories converged in China and competed for recognition, which rapidly increased the theoretical research and practical use of PD in mainland China.

The period from 2004 to 2014 can be regarded as the period of prosperity with respect to the development of PD in mainland China. The PD community in mainland China widely promoted and developed the practice and methodology of PD and gradually began to explore the possibilities of its localization and adaptation. At this stage, the form of campus PD was developed in universities from the bottom up in mainland China. It has since demonstrated rapid development in Chinese universities and has become one of the most effective, lively, and expressive ways of promoting mental health education.

Additionally, as shown in **Table 1**, PD research in China mainly started at the beginning of the 21st century and particularly after 2005. This upsurge in PD research continues to the present day. These studies mainly summarized the therapeutic techniques of PD and the effectiveness of treatment for specific populations (including patients with specific mental disorders and specific social stratum). Among them, most studies have focused on patients in psychiatric hospitals and college students.

**Table 1:** Articles on psychodrama published from 1980 to 2017 in the CNKI.

Year of publication	Number of papers	Comments
1980–1990	1	Word “psychodrama” appears in the title
1991–2000	7	
2001–2005	27	
2006–2010	98	
2011–2017	236	

*Information as of December 31, 2017.*

### Period of New Development

After 2004, intensive training in practical PD skills was provided in China; however, no unified nationwide authoritative organization existed to develop a regular training program. In 2014, during the second session of the Chinese conference on group counseling and group therapy, the Division of Group Counseling and Group Therapy of CAMH conducted a meeting, where they adopted a resolution for the establishment of the Group of Psychodrama. This organization sought to “further promote the training and use of PD, strengthen the study, supervision and ethics training in PD, hold PD international seminars at the appropriate time, continue encouraging Chinese psychodramatists to participate in the examination of the ABEPSGP, and attempt to establish China’s own evaluation and examination system in the future” (Sang, 2015). At the same time, combined with the Chinese culture and characteristics, the use of psycho-scene-drama was vigorously promoted in schools, enterprises, and different workplaces.

The development of PD in mainland China could subsequently be ensured and maintained by an authoritative academic organization, and its development entered a new phase. Therefore, this event can be considered a critical point between the period of prosperity and the period of new development.

In summary, the above mentioned four periods are divided according to the crucial events involved in the spread and development of PD in mainland China. The first crucial event was in 1947, when Ding Zan came into contact with PD in the United States and introduced it to China. The second crucial event was in the 1980s. Chen (1982) and Li (1988) introduced PD as a psychotherapeutic method for treating mental illness in academic journals and thus introduced PD to the Chinese field of psychiatry. Contact with PD was opened again after an interruption of more than 30 years. The third crucial event occurred in 2004, when Gong Shu, Katherine Hudgins, Lai Nianhua, and other trainers began coming to China to conduct systematic and continual PD technical training. The popularization and development of PD in mainland China entered a stage of rapid development. The fourth crucial event was the establishment of the Group of PD in the Division of Group Counseling and Group Therapy of CAMH in 2014. Since then, the development of Chinese mainland PD has become more normative and orderly.

## Development of Different Theoretical Branches of Psychodrama in Mainland China

The popularization of PD in mainland China has undergone a four-stage process. However, discussing all branches of PD together could lead to overgeneralization. In fact, since Zerka Moreno first promoted PD techniques, many psychologists have further revised and developed PD theory according to their own clinical practice experience and distinctive theoretical concepts, which resulted in the development of different branches of PD. These theories were introduced in mainland China in different ways and influenced Chinese psychologists’ understanding of the concept of PD. The most widespread PD models in China are Yi Shu PD and the TSM. Furthermore, Chinese psychodramatists created an innovative type of PD, known as campus PD, which was used in mental health education for college students and thereby led to support theoretical research and development of practical PD models.

### Yi Shu Psychodrama

Yi Shu PD, developed by Gong Shu, is a therapeutic PD concept, which is the result of over 30 years of practice in the fields of art therapy, Gestalt therapy, PD, and traditional Chinese medicine. Yi means “change”, Shu means “the way”, the “art”, or “the dao”. Yi Shu simply means “the art of living with change” (Gong, 2012:57). The publication of the monograph “Yi Shu: The Art of Living with Change-Integrating Traditional Chinese Medicine, Psychodrama and The Creative Arts” in the United States in 2003 marked the official formation of the theory of Yi Shu PD.

Yi Shu is a form of group psychotherapy that can also be used in individual therapeutic sessions. Its purpose is to “open the energy that is blocked by individuals, a group, or many groups” (Gong, 2007:107). The philosophical foundation of Yi Shu lies in the concept of “the organism as a whole”, as well as spontaneity and creativity. Yi Shu uses the generating and restricting natures of the five elements in Chinese philosophy (Wu Xing: water, wood, fire, earth, metal) to treat human emotions and their related energy imbalances in the body. The visible physical body is yang in nature, whereas the invisible energy body is yin in nature. Yin and yang coexist at all times, influencing and transforming into each other. Emotions belong to the invisible energy body, whereas physical symptoms belong to the visible physical body. Emotional imbalances often affect the physical body. In the healing process of Yi Shu, the energy body and the physical body are simultaneously healed (Gong, 2008).

The basic procedure of Yi Shu is as follows: (1) Sitting still to practice Qigong (a system of Chinese physical exercises and breathing control), opening the conception vessel and governor vessel by relaxing, breathing, and imagining, and starting the energy flow of the human meridians. In theory, sitting still to practice Qigong can help people remove trivial matters from their minds and reach the deep potential that they were endowed with at birth. (2) Using music to promote breathing and allowing the body to swing. Through soft meditation music, or trance-inducing drums, the inner rhythm of the individual is regulated and the obstructed energy is opened. (3) The group members paint on Xuan paper (a soft rice paper with texture) with a brush. Chinese painting is used to cultivate spontaneity and creativity, and enables individuals to present their inner experience. (4) By acting and role playing each image and coloring in the pictures, the client can touch the feelings and meanings hidden inside them. Sometimes, during the performance, the director or the protagonist will be asked to complete some sentences, such as “I feel…,” “I need…,” and “I am afraid....” The group members are selected as auxiliaries to act out these colors and images. The auxiliaries must pay attention to the protagonist’s voice, action, speech, and the positions and relationships presented by the protagonist. The protagonist sits among the audience to watch the performance, as if watching the story of others. The pattern, action, and sound of the role form the gestalt of the protagonist’s spiritual reality. (5) Through PD procedures, such as role reversal, doubling, mirroring, and soliloquy, the client is assisted in accomplishing further exploration, accompanied by the release of energy blockages in the body and blood. Generally, the steps of Yi Shu are mostly the same form as in PD, such as warm-up, action, and sharing. Yi Shu works well with and naturally compliments classical PD with intermodal applications of Qigong, Chinese painting, shamanic music, and the use of the acupoints on the human body to adjust the body’s energy to achieve harmonious functionality of the individual’s viscera and emotions (Gong, 2007:107–109).

Gong Shu is the first psychodramatist to establish a connection with Chinese psychologists; therefore, her PD theory was introduced relatively early and expeditiously became rooted in mainland China. Moreover, the features of oriental thinking mode and traditional Chinese medicine concepts were integrated into Yi Shu PD, which also made it more acceptable and applicable for practice in China. Numerous Yi Shu PD workshops were conducted in major cities in China, and the International Center for Yi Shu Body and Soul Therapy at Suzhou University was established. After the establishment of the center, some scholars conducted a series of research projects on the therapeutic effects and methods of Yi Shu PD; for example, in patients experiencing depression or emotional letdown who are in the process of learning to regulate their emotions and those who have experienced adolescent sexual trauma. These studies validated the therapeutic effect of PD to a considerable extent (Ji and Wang, 2012).

### Therapeutic Spiral Model

TSM, developed by an American PD trainer Katherine Hudgins, is an integrated method of experiential psychotherapy that combines classical PD with advances in clinical psychology and trauma work to provide the containment required to prevent retraumatization (Hudgins et al., 2000).

Because many trauma survivors often experience internal confusion and interpersonal distress metaphorically akin to a tornado, TSM chooses a spiral as a therapeutic model to provide an alternative dimension to the incontrollable energy of tornadoes. Clients learn to move up and down the spiral of their own needs instead of being torn apart by the chaos of the tornado (Deng et al., 2009:84).

The therapeutic spiral image in the TSM is divided into three categories: energy, experience, and meaning (Hudgins, 2003). The TSM comprises six safe modes: (1) positing and embodying the observed ego (OE); (2) circle of safety; (3) spectrogram; (4) action sociogram; (5) circle sociometry; and (6) mastermind art activity. The six safe modes help trauma survivors express themselves. In addition, body double, containing double, prescription character, and other techniques in the TSM made it safe for clinical practice, increased the therapeutic effects of action treatment for trauma, and prevented clients from experiencing retraumatization (Sang, 2009).

The following are the features of TSM: (1) The drama begins by seeking the power of the clients themselves. If the clients are not sufficiently strong, the director attempts to make them more powerful. Some of the power comes from prescriptive roles, such as body doubles, or from interpersonal interactions acted out by the selected auxiliaries. (2) Supporting wound healing is conducted with a containing double. The containing double provides clients with intense feelings of security, stability, and tolerance, and helps them establish a framework to understand the connotations when they experience strong emotions. (3) The expression of emotion is controlled spirally. With the help of the body doubles and containing doubles, the director alters the mood of the protagonist spirally and slowly. TSM keeps the clients aware of their emotions, and maintains effective and reasonable control of their emotional responses in the catharsis process (Deng et al., 2009:95–97). Unlike classical PD, the steps and psychological impact of the TSM were adjusted, and more new practical applications of PD techniques were developed, which resulted in reasonable and conscious control of clients’ psychological and behavioral degradation processes (Liu, 2007).

In May 2004, Hudgins visited Nanjing University for the first time to provide professional training on the TSM. After the 2008 Sichuan earthquake, numerous psychologists in China rushed to the areas affected by the disaster and used various forms of group psychotherapy to deal with victims’ post-traumatic symptoms. At this point, the wide use of the TSM and its unique method of safely approaching trauma began to attract the attention of Chinese psychologists. From 2008 to 2010, Hudgins visited Chongqing and several districts of Sichuan with a group of Chinese psychodramatists to teach PD skills to local mental health teachers in primary and secondary schools and train them to use PD techniques flexibly with students to treat the trauma caused by the disaster. As a unique and safe mode of healing, the TSM has been widely used in the clinical practice of trauma treatment in China. It has been commonly used in areas with groups experiencing the negative aftermath of disasters, among public security forces and firefighting forces, at campus stress events, and in enterprise staff support interventions.

### Campus Psychodrama

Campus psychodrama, also known as campus psycho-scene-drama, is based on the Chinese native culture and national traits. It imbibes the essence of expressive art, including drama, opusculum, PD, and musical. It is a type of “expressive arts in action” developed in Chinese mental health education practice (Deng et al., 2009:171). Using PD techniques, such as role-playing, campus PD reproduces psychological activities and conflicts and enables participants to recognize and solve problems, either by themselves or with the assistance of other participants. Campus PD scripts are written based on the common problems faced by students, allowing them to intuitively learn and understand psychological knowledge in the process of writing, rehearsing, and performing. It illustrates a psychological story in real life and has educational significance for the audience (Yu, 2013). Campus PD is popular among Chinese students because its techniques, such as scenery, dialog, soliloquy, narration, and role reversal, vividly present different aspects of campus life.

In addition to the typical characteristics of PD, such as spontaneity and creativity, campus PD is also thematic and educational. First, it is thematic because it usually focuses on certain topics, such as interpersonal communication, stress response, parent-child education, and self-growth. The process of determining the theme is also an effective warm-up process, which can eliminate cognitive impedance. Moreover, if the group selects a non-thematic performance, it becomes a theme in itself, that is, a free and spontaneous performance of the group’s feelings by discussing and performing topics of common concern. Second, campus PD is educational because it emphasizes educational inspiration and moderate guidance, and plays an exemplary role through situational performance. For example, campus PDs regarding moral education, interpersonal communication, and love frustration can make actors and audience think deeply and find solutions in their own lives (Sang, 2017).

Campus PD draws on many PD techniques; however, it is different from PD in the following aspects: (1) PD promotes dealing with mental health problems at an individual level, whereas campus PD is aimed at dealing with the common mental health education issues faced by a particular group of people with certain characteristics. (2) PD is used for psychological counseling and therapy for serious mental health problems and mental disorders, whereas campus PD is usually used for general mental health education for ordinary people. (3) PD requires qualified directors and auxiliaries who receive considerable professional training to help the protagonist finish the psychotherapy; however, the director of campus PDs do not require the strict professional training of a PD director, and the actors are usually non-professional group members. The audiences of campus PDs are typically more homogenous than those of PDs (Deng et al., 2009:193–194).

Campus PD gained attention in 1999 at a special workshop of the Sixth Annual Meeting of the College Students’ Psychological Consultation Specialized Committee of the CAMH. As a new form of mental health education, it became widely popularized in primary and secondary schools and universities in mainland China. In many universities, campus PD competitions became part of annual campus culture projects (Yu, 2013). Some Chinese researchers carried out empirical research to explore the mental health education effect of campus PD on the topics of interpersonal communication barriers, adaptation of college freshmen, and social responsibility training (Huang, 2015; Du et al., 2017; Qian, 2017).

The method of psycho-scene-drama in China has gradually been extended from school education to prisons, the army, enterprise training, and other fields. Wu (2016) used psycho-scene-drama to study the intervention of cognitive emotion regulation among prisoners, which showed that psycho-scene-drama can be used as an effective form of psychological treatment in prisons.

## Discussion

Since its introduction in China, PD has been widely used in all types of psychological counseling and treatment, mental health education, and group cohesion and interpersonal behavior training in business settings and other fields due to its emphasis on interpersonal groups, dynamic systems, self-metaphorization, and other characteristics considered vital in Chinese culture and has achieved highly successful results. Because PD was studied and applied in Taiwan before it was in mainland China, Taiwanese clinical practice experience and academic research are valuable for mainland scholars. For example, Taiwanese scholar Lai and Tsai (2014) reported in a quantitative empirical study on the process of PD that the final sharing and discussion often took more time and energy in a Chinese cultural context than it did in the Western context. Feedback from the direct participants of PD and the audience also suggested that they felt they benefited most from sharing and discussion. Many possible explanations existed for this phenomenon but cultural characteristics were certainly a factor. This may reflect the need for slow pace when working within the Chinese culture. Possibly influenced by the suppressive and indirect manner of communication among the Chinese, participants in this culture tend to warm up slower in group activities than their western counterpart. On the other hand, clients may exhibit eagerness to share before finishing the session. This has prompted mainland PD workers to focus more on sharing and the cultural differences in practice.

This overview of the development of PD in mainland China illustrates the “many streams, one river” feature, which was specifically caused by factors, such as commercialization and uneven geographical development. Therefore, although the dissemination of multiple theoretical branches in the initial period of the development of PD was disintegrated, the general context of the development of mainstream PD in mainland China is clear. That is, from a temporal perspective, it can be divided into four stages of development, and from a spatial perspective, it originates in Nanjing and Suzhou and gradually spread to all parts of the country. This can be seen from the composition of the founding committee of the Group of Psychodrama of the Division of Group Counseling and Group Therapy of CAMH. PD scholars in mainland China have now begun to have multiple connections with international PD groups. At present, 13 have been accredited by the ABEPSGP. And many mainland scholars have been certified by the British Psychodrama Association. Mainland scholars, such as Wang Er-dong, have conducted continuous PD training in Malaysia, extending PD training and promotion work to other countries. Therefore, we believe that PD in mainland China has entered a stage of steady development.

Due to its high cultural adaptability, the application prospects of PD in China are broad, as with the Chinese use of Campus Psychodrama carried out in primary and secondary schools and universities, and its application in human resource management and prisoners’ behavior correction. However, we must also acknowledge that although the Group of Psychodrama in CAMH has been established, further standardization of PD training and supervision is still a goal that PD workers in mainland China must strive to achieve. The gap between the levels of academic research on PD in mainland China and Taiwan and other international research is still obvious. This has prompted psychologists in mainland China to conduct research on PD in Chinese cultural settings and learn from the studies of Taiwanese academia to better serve the clinical practice of PD in mainland China.

## Author Contributions

Z-qS and H-mH reviewed literature and wrote the manuscript. AB translated the manuscript. Z-qS and YW revised the follow-up versions of the manuscript.

## Conflict of Interest Statement

The authors declare that the research was conducted in the absence of any commercial or financial relationships that could be construed as a potential conflict of interest. The reviewer ST and handling Editor declared their shared affiliation at the time of the review.
